# Arithmetic Skill May Refine the Performance of Individuals with High Math Anxiety, Especially in the Calculation Task: An ERP Study

**DOI:** 10.1038/s41598-019-49627-7

**Published:** 2019-09-16

**Authors:** Bijuan Huang, Xiaomeng Zhao, Hongxia Li, Weixing Yang, Shuang Cui, Yaru Gao, Jiwei Si

**Affiliations:** School of Psychology, Shandong Normal University, Jinan, China

**Keywords:** Human behaviour, Emotion, Electrophysiology

## Abstract

As a global phenomenon, the theme of math anxiety has received increasing attention. The present study aimed to investigate the relationship between math anxiety and performance and determine the role of arithmetic skill in two different tasks. Fifty-seven college freshmen were recruited to perform a comparison task and a calculation task. Only main effect of arithmetic skill was found on the behavioral level. In the comparison task, participants with high math anxiety (HMA) showed faster latencies and greater amplitudes of N1 and longer P3b latency relative to their counterparts with low math anxiety (LMA). Number, as a negative stimulus, occupied attentional resources and delayed the speed of cognitive processing for individuals with HMA. Furthermore, among those with HMA, individuals with high arithmetic skill showed larger amplitudes and shorter latencies of P2 compared with those with low airthmetic skill in the calculation task. Thus, arithmetic skill could refine the performance efficiency of individuals with HMA, especially in the calculation task. These results suggest that educational interventions emphasizing control of negative emotional responses to math stimuli will be more effective when considering different populations of mathematically competent individuals.

## Introduction

Recently, a considerable literature has grown up around the theme of the math anxiety–performance link. As a global phenomenon^1^, math anxiety is commonly defined as a negative emotional reaction (tension, apprehension or fear) when dealing with numbers or math-related situations, which disrupts math performance^2,3^. Individuals with high math anxiety (HMA) tend to avoid courses related to math in both high school and college, receive lower grades, hold a negative self-concept and attitude toward math, and have lower confidence and lower motivation^2–6^. These negative impacts lead to adverse effects on career choice, which is of particular importance for future long-term professional success^7^. Consequently, it is meaningful and critical to investigate the relation between math anxiety and performance.

## Inconsistent Findings of Math Anxiety–Performance Link in Behavioral Research

As mentioned above, math anxiety interferes with math performance. In behavioral research, however, the findings are contradictory. Some researchers reported the effect of math anxiety on math performance or numerical tasks, whereas other studies found no evidence of this effect. For example, Colomé (2018) revealed no group difference in a nonsymbolic comparison task^8^, in contrast to the study of Dietrich *et al*.^9^. The negative effect of math anxiety may be dependent on task complexity, known as anxiety**–**complexity effect, where the effect of math anxiety was only observed in complex problems^3,10–14^. However, Maloney and collaborators (2010) reported that for error rate, a group difference was also found in counting^15^.

The main predictions of processing efficiency theory and attentional control theory are that anxiety impairs processing efficiency (the effectiveness of performance divided by the effort or resources spent in task performance, e.g., reaction time (RT)) more than performance effectiveness (the quality of performance, e.g., accuracy)^16,17^. More specifically, negative effects of anxiety are predicted to be significantly greater on efficiency than on effectiveness^17^. Young *et al*. (2012) indicated people with HMA showed marginally lower accuracy in complex tasks but no difference in RT^7^. However, Núñez-Peña and Suárez-Pellicioni (2014) found a group difference in RT in a single-digit number comparison task^18^. Some other studies suggested the effect was only found in flawed score and event-related potentials (indicator of processing efficiency) for larger-split problems^19^.

Therefore, the inconsistency of behavioral data may be due to different tasks used, or subtle differences were showed in the way that people with HMA and with low math anxiety (LMA) performed in numerical tasks, which could not be perceived in behavioral studies and was distinguished by differential recruitment of neural resources^20^.

## Findings of Math Anxiety–Performance Link in Psychophysiological Research

Overall, from the perspective of psychophysiology, studies highlighting the effect of math anxiety have increased. In numerical cognition, some event-related potentials (ERPs), such as N1, P2 and P3b, are differentially associated with math anxiety^21,22^. P3b, a positive peak that appears approximately 300 ms after the stimulus is presented, is considered to be sensitive to cognitive processing. Its amplitude is linked to the amount of attentional resources allocated to the stimulus, and its latency is related to the stimulus evaluation time^19,23^. Suárez-Pellicioni *et al*. (2013) explored the effects of math anxiety on simple arithmetic processing and found HMA elicited more positive amplitude and longer latencies of a late positive component (P600/P3b) in large-split solutions^19^. They concluded that individuals with HMA had difficulty suppressing irrelevant information processing.

In addition, Núñez-Peña and Suárez-Pellicioni (2014) found a P2 component in a comparison task. Larger P2 amplitude was showed in HMA individuals compared with LMA peers, suggesting a less precise representation of numerical magnitude for HMA individuals^18^. P2, a positive peak that appears 150-250 ms after the stimulus is presented, is considered to be related to attentional resources allocated to negative stimulus in numerical cognition^23^. The P2 in the frontal region may reflect an inhibitory ability to unrelated stimuli^23,24^. Chang and colleagues (2017) found reduced fronto-parietal attention network activations in those with LMA compared to those with HMA, suggesting that simple arithmetic was not simple for those with HMA^20^. Since neuroimaging is a valuable way of assessing processing efficiency, it is better to combine neuroimaging and behavioral data^17,25^. Lyons *et al*. (2012) concluded that increased activation in fronto-parietal regions mitigated deficits in math-related performance for HMA when simply anticipating doing math^26^. Although increasing studies focused on the neural correlates, they varied greatly in the method and task (from simple numerical to complex arithmetic)^27^. We adopted two kinds of tasks in this study, a numerical comparison task and a calculation task.

## Math Anxiety–Performance Link: The Role of Arithmetic Fluency

Some individual factors, such as motivation or arithmetic skill, may affect the relationship between math anxiety and performance^4,6,28^. Lyons and Beilock (2012) indicated that not all individuals with math anxiety performed equally poorly in math and suggested that control of negative emotional responses to math stimuli should take a population of mathematically competent individuals into account^29^. The deficit of math anxiety may be due to math ability difference rather than math anxiety itself^27^. Very few studies have explored how basic arithmetic ability (indexed with arithmetic fluency^30^) affected math performance, especially within the HMA group. Fluency with simple arithmetic, typically achieved in early elementary school, is regarded as one of the building blocks of mathematical competence^20^. Thus, we postulated that arithmetic skill has a different effect on the math anxiety–performance link. As a premeasure (see Supplementary Fig. S8), individuals with a high level of arithmetic skill and with a low level of arithmetic skill exist both in the HMA group and the LMA group. Moreover, no group difference in math anxiety was found in students with high arithmetic skill or with low arithmetic skill.

The objective of the present research was to investigate neural correlates on the math anxiety–performance link and to determine the potential roles of basic arithmetic skill in two tasks. Four groups with different levels of math anxiety and arithmetic skill were chosen to perform a numerical comparison task and a calculation task. With the aid of electroencephalography (EEG), behavioral and electrophysiological data were collected. The effect of arithmetic skill was expected to be observed on behavioral performance. We expected to find the effect of math anxiety in the early component (e.g., N1, P2) and the late component (e.g., P3b). Students with high arithmetic skill, who need less working memory resources, were expected to be more fluent in numerical processing. Consequently, the moderating role of arithmetic skill was expected to be found in all ERPs. In addition, group differences of math anxiety were observed for P3b latency and amplitude in parietal sites^19^. RT was also expected to be positively related to P3b latency, and negatively related to P3b amplitude. This study offers some important insights into the theme of the math anxiety–performance link and provides some suggestions for math education or intervention for controlling math-related negative emotional responses in school.

## Material and Methods

### **Participants**

Fifty-seven college freshmen were recruited in this experiment, which were selected from a large sample of 1038 students from Shandong Normal University who were administered the Chinese adaptation of the Revised-Mathematics Anxiety Rating Scale (R-MARS)^31^ and the French Kit^30^. Volunteers with scores within the first or fifth quintile on R-MARS and the French Kit (LMA: ≤ 20% on R-MARS; HMA: ≥ 80% on R-MARS; Low arithmetic skill (LAS): ≤ 20% on the French Kit; High arithmetic skill (HAS): ≥ 80% on the French Kit) were invited to participate in subsequent experiments. Considering balancing their gender and major in four groups, there were only 64 subjects left. Among these, 3 subjects did not participate in subsequent experiments and were excluded. Four additional participants were excluded due to low accuracy and excessive artifacts in the EEG data, with a final sample size of 57. Participants with different levels of arithmetic skill and math anxiety were divided into four groups: 14 students with LAS & LMA (7 females, mean age 18.07 ± 1.07), 15 students with LAS & HMA (8 females, mean age 18.33 ± 0.49), 13 students with HAS & LMA, (5 females, mean age 18.15 ± 0.38), and 15 students with HAS & HMA (9 females, mean age 18.13 ± 0.64) (more detailed information about the four groups is shown in Table 1). All students were right-handed, had normal or corrected-to-normal vision, confirmed that they did not have any psychiatric or neurological disorders, and were asked to sleep as adequately as possible on the night before. All procedures of the study were in accordance with the ethical standards of the institutional and/or national research committee and with the 1964 Helsinki Declaration and its later amendments or comparable ethical standards. The ethics committee of the Shandong Normal University approved the study. All participants gave written informed consent before the experiment started and were compensated with a small gift for participation.

**Table 1:** Demographic Characteristics of Participants.

	LAS		HAS		Group Difference
	LMA	HMA	LMA	HMA	
*N*	14	15	13	15	—
Sex (M/F)	7/7	7/8	8/5	6/9	—
Age (*SD*)	18.07(1.07)	18.33(0.49)	18.15(0.38)	18.13(0.64)	—
MA (*SD*)	27.00(1.71)	63.47(9.95)	25.92(2.18)	58.60(5.19)	
AS (*SD*)	73.36(6.10)	73.20(4.52)	138.00(11.32)	134.00(11.66)	N.s.
TAS (*SD*)	12.21(4.19)	16.40(4.24)	12.08(4.65)	20.87(5.85)	**
STAI-S (*SD*)	32.43(9.10)	36.47(5.54)	33.08(5.95)	40.07(8.28)	**
STAI-T (*SD*)	34.50(10.64)	40.67(7.27)	36.08(5.78)	46.20(9.56)	**

Note. LAS: low arithmetic skill; HAS: high arithmetic skill; LMA: low math anxiety; HMA: high math anxiety; AS, MA, TAS, STAI-S and STAI-T are the means and standard deviations (in black bracket) of arithmetic skill, math anxiety, test anxiety, state anxiety and trait anxiety, respectively; N.s.: Not reaching a significant level; **p < 0.01, *p* value was obtained using multivariate analysis of variance (MANOVA) and suggested a significant difference between LMA and HMA in TAS, STAI-S and STAI-T.

Correlations between math anxiety, test anxiety, state anxiety and trait anxiety were all significant (all *P*s < 0.05) (see Supplementary Table S1). Therefore, these three variables were controlled in subsequent analysis. The current sample size was adequate to detect an effect of *f* = 0.25 (*d* = 0.5) at *α* = 0.05, for an *F* test (repeated-measures analysis of variance (rmANOVA) within-between interaction, with four between-subject and two within-subject conditions) (power level of 0.88 for post hoc analysis by G*power 3.1 software). The sample sizes of recent studies that have investigated math anxiety with ERPs ranged from 13 to 28^8,19^. Accordingly, the sample number of each group was appropriate.

### **Materials**

#### R-MARS

The Chinese adaptation of R-MARS^32^ consists of 21 items representing various math-related situations faced by students. Participants were asked to rate the level of anxiety on a 5-point Likert scale from 1 to 5. The Cronbach’s α coefficient of the scale in the current study was 0.98.

#### The French Kit

The French Kit^30^ is used to assess basic arithmetic skill with addition, subtraction and multiplication, administered by paper and pencil. It consists of four subtests. Two subtests are addition, and the other two are subtraction and multiplication. Each subtest contains 60 items, and participants are given only 2 minutes to solve problems as quickly and accurately as possible. The total number of correctly solved problems is calculated.

#### Test Anxiety Scale (TAS)

The TAS^33^ is a 37-item scale to assess the attitudes about tests and the feelings before and after tests. Participants were asked to answer *yes* or *no* to each question. The Cronbach's α coefficient of the scale in the current study was 0.82.

#### State-Trait Anxiety Inventory (STAI)

The Chinese adaptation of STAI^34,35^ consists of 40 items and is used to assess the levels of state and trait anxiety. For each item, participants answered on a Likert scale from 1 to 4. The Cronbach’s α coefficient of the state scale in the current study was 0.88, and that of the trait scale was 0.89.

### **Procedure**

The experiment was conducted in a quiet room. Participants were asked to perform two different tasks, a numerical comparison task and a calculation task. They were presented using E-prime 2.0.8.22 (Psychological Software Tools, Pittsburgh, PA) in a blocked fashion. The number of trials in each task was 88, including 8 practical trials and 80 experimental trials. A black stimulus was rendered on the white background in Times New Roman (for more detailed information about stimuli, see Supplementary Table S3). The order of the tasks was randomized across participants in an equivalent way for the four groups.

#### Numerical comparison task

**Figure 1:**
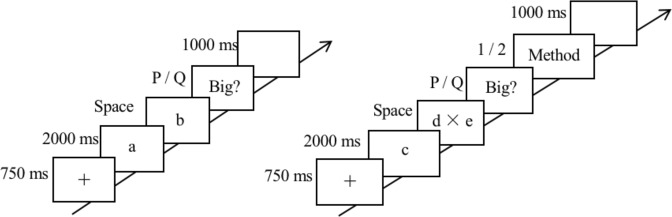
The structure and timing of one trial. Left, timing of one trial in the comparison task block. a: The first number. b: The second number. In the comparison task, subjects were asked to compare b with a, and decide whether b was bigger or not by pressing “p” or “q”, respectively. Right, timing of one trial in the calculation task. c: The solution. d and e: The first operand and the second operand of a multiplication problem, respectively. In the calculation task, subjects were asked to decide whether the result of d × e was greater or not by pressing “p” or “q”, respectively. Then, they indicated the method they used to solve the problem by pressing “1” (approximate calculation) or “2” (exact calculation), respectively.

Each trial began with a 750-ms fixation cross in the center of the screen, followed by the presentation of the first number (e.g., a) for 2000 ms. Then, the second number (e.g., b) appeared, and participants were asked to decide whether b was greater than a. During this period, they were asked to press the space bar as soon as they made the judgment. Then, a screen saying ‘Big?’ was present. Participants were asked to press the “p” or “q” button on the keyboard to indicate whether b was greater than a or not, respectively. Trials were separated by a black screen lasting 1000 ms (For the timing of one trial, see Fig. 1).

#### Calculation task

This task was an arithmetic verification task. Each trial started with a fixation cross in the center of the screen lasting 750 ms. A solution (e.g., c) was then presented for 2000 ms, followed by the presentation of an operation (e.g., d × e). During this period, participants were asked to decide whether the result of d × e was greater than c or not and pressed the space bar as soon as they had made the judgment. Then, a screen with the word “Big?” appeared, and participants were asked to press the “p” or “q” button on the keyboard to indicate their answers. After responses, participants selected the method they used to solve the problem, followed by a blank screen for 1000 ms. Two methods of calculation were provided for the participants, namely, approximate calculation (press “1” on the keyboard) and exact calculation (press “2” on the keyboard) (The timing of one trial can be seen in Fig. 1).

### **Electroencephalographic recordings**

In a dark and sound-attenuated room, participants were seated in a comfortable chair, and the stimuli were presented on a monitor located approximately 80 cm in front of the participant. EEG data were recorded using a Brain Production system (BrainVision Recorder 1.20, Brain Products GmbH, Germany, http://www.brainproducts.com) with a cap of 63 electrodes positioned according to the extended 10/20 system and connected to a QuickAmp amplifier. The reference electrode was placed at the midpoint between Fz and Cz, and ground was located at AFz. The horizontal and vertical electrooculograms were recorded with electrodes placed at the outer canthi and below the right eye, respectively. A bandpass filter was set from 0.05 to 100 Hz, with a sampling rate of 1000 Hz. The impedances of all electrodes were kept below 10 kΩ.

Analyzer 2.1 (BrainVision Analyzer 2.1.0, Brain Products GmbH, German, http://www.brainproducts.com) was used to process the offline signals. After a visual inspection to discard saccades, all offline signals were referred to the average value of the left and right mastoid, and the original reference electrode was renamed FCz. The data were digitally filtered (low-pass filter of 35 Hz, zero-phase, and slope of 24 dB/oct). After that, eye-related artifacts, such as blinks, were detected and corrected by independent component analysis. Then, the data were segmented into 1200-ms epochs that included a 200-ms prestimulus baseline and 1000 ms after the onset of the second number in the comparison task or the operation in the calculation task. In that procedure, only trials with correct responses were maintained. Participants with a number of trials segmented less than 50 were excluded (*N* = 4). After a baseline correction from −200 to 0 ms (with 0 marking the time point of stimulus presentation) was conducted, epochs containing voltage changes that exceeded ±80 μV at any electrodes were excluded from the analysis. The remaining epochs were separately averaged for each group in each task. The average trial number for the four groups was 70.63 (*SD* = 7.50) in the comparison task and 64.86 (*SD* = 7.32) in the calculation task. The number of epochs contributing to the participant averages did not significantly differ in the math anxiety (MA) (*p* > 0.05) but substantially in the arithmetic skill (AS) or task (*ps* < 0.05). From within-subject averaged waveforms, peak latency and amplitude were extracted. Previous studies have revealed the effects of MA on N1, P2 and P3b^18,19,21^. Accordingly, the peaks of N1, P2, and P3b were determined as the maximum point 80–120 ms, 150–250 ms, and 250–500 ms following stimulus presentation, respectively. ERPs were analyzed during epochs at eighteen electrodes, as shown in Supplementary Fig. S1. Based on the typical distribution of these responses, nine fronto-central electrodes (F3, Fz, F4, FC3, FCz, FC4, C3, Cz, and C4) were selected for statistical analysis of N1 and P2; and nine parietal-occipital electrodes (P3, Pz, P4, PO3, POz, PO4, O1, Oz, and O2) were selected for statistical analysis of P3b.

### **Data analyses**

For behavioral data, accuracy (ACC, the percentage of correct responses) and RT (reaction time for correct responses) are frequently used to assess performance effectiveness. Flawed score is another important parameter, which is the sum of the proportion of errors and the proportion of extreme RT values (determined by stem-and-leaf)^13,19^. Flawed score is considered as a measure of processing efficiency and is devised to detect difficulties in processing. According to Faust *et al*. (1996) and Suárez-Pellicioni *et al*. (2013), the extreme RTs were retained, and the RTs of incorrect trials were excluded. In the behavioral level (RT, ACC, and flawed score), MANCOVA was performed for the comparison and calculation task separately, with AS and MA as between-subject factors, and test anxiety, state anxiety and trait anxiety as covariates. Bonferroni correction was used for post hoc multiple comparisons (for descriptive statistics, see Supplementary Tables S2 and S3).

For the neurophysiological data, four-way rmANOVAs with area (frontal, fronto-central, and central/parietal, parietal-occipital, and occipital), hemisphere (left: F3, FC3, C3/P3, PO3, O1; midline: Fz, FCz, Cz/Pz, POz, Oz; and right: F4, FC4, C4 / P4, PO4, O2) as within-subject factors, AS (LAS vs. HAS) and MA (LMA vs. HMA) as the between-subject factors, and test anxiety, state anxiety and trait anxiety as covariates were performed for three components of interest in the comparison and calculation task separately. Greenhouse–Geisser corrections were applied to reduce the type I error rate when necessary. Bonferroni correction was used for post hoc multiple comparisons.

## Results

### **Behavioral results**

#### **The HAS group merely performed faster in the comparison task**

**Figure 2:**
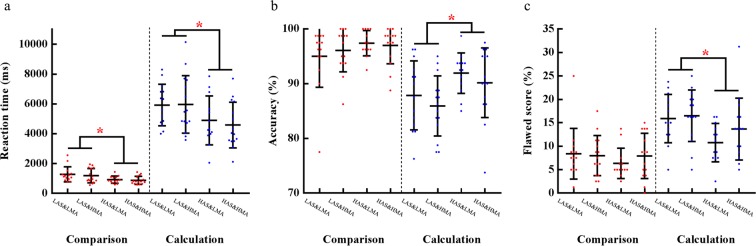
The descriptive statistics of ACC, RT, and flawed score for all four groups. (**a)** For RT, LAS > HAS both in the comparison task and calculation task. (**b**) For ACC, LAS < HAS only in the calculation task; (**c**) Flawed score of LAS > flawed score of HAS only in the calculation task. A dot in each bar represents the data point of one participant. The red dots illustrate the data of the comparison task, and the blue are the calculation task. Note: * *p* < 0.05 indicates significant difference; error bars indicate standard error of the mean; values of covariates: state anxiety = 35.65, trait anxiety = 39.56, and test anxiety = 15.56 (the same below).

Only in RT did MANCOVA reveal a significant main effect of AS [*F*(1, 50) = 9.37, *p* = 0.004, η_p_^2^ = 0.16]. Compared to the LAS group (1233.00 ms), significantly shorter RT was found for the HAS group (893.44 ms) (see Fig. 2a–c). No other effects were found.

#### **High arithmetic skill improved performances in the calculation task**

The main effect of AS was significant in RT [*F*(1, 50) = 7.40, *p* = 0.009, η_p_^2^ = 0.129], ACC [*F*(1, 50) = 6.34, *p* = 0.015, η_p_^2^ = 0.11], and flawed score [*F*(1, 50) = 6.97, *p* = 0.011, η_p_^2^ = 0.12]. The HAS group (RT: 4717.76 ms; ACC: 90.96%; flawed score: 12.19%) performed better than the LAS group (RT: 5975.43 ms; ACC: 87.01%; flawed score: 16.22%) (see Fig. 2a–c). No other effects were found.

### **Electrophysiological results**

#### **Deficits of math anxiety in the comparison task**

**Figure 3:**
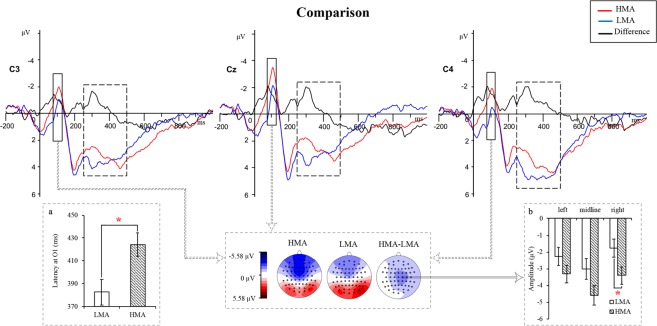
Grand-average waveforms of N1 (highlighting with solid rectangle) and P3b (highlighting with dashed rectangle) for the HMA group and LMA group at the C3, C4 and C4 sites in the comparison task. (**a**) P3b difference of MA. The latency of the HMA group was longer than that of the LMA group. (**b**) N1 difference of MA. As suggested, compared with the LMA group, amplitudes in HMA group were more negative at right sites. Additionally, the scalp topographies of the N1 component showed the group difference in the 80–120 ms window. *p < 0.05.

*N1*. **For latency**, a significant main effect of MA was found [*F*(1, 50) = 5.93, *p* = 0.018, η_p_^2^ = 0.11], indicating that the latencies of N1 in LMA individuals (109.59 ms) were longer than that in their HMA peers (101.96 ms). The interactions of area × hemisphere [*F*(3.21, 160.30) = 4.10, *p* = 0.007, η_p_^2^ = 0.08] reached the level of significance. **For amplitude**, analysis revealed a significant main effect of hemisphere [*F*(2, 100) = 6.78, *p* = 0.002, η_p_^2^ = 0.12]. Amplitudes at the midline sites (−3.79 μV) were more negative than those at left sites (−2.79 μV) and right sites (−2.59 μV). Significant interactions of area × hemisphere [*F*(3.22, 160.95) = 3.96, *p* = 0.008, η_p_^2^ = 0.07] and MA × AS [*F*(1, 50) = 4.51, *p* = 0.039, η_p_^2^ = 0.08] were observed. Post hoc tests revealed that in the HAS group, N1 amplitude in HMA individuals (−4.47 μV) was higher than that in their LMA counterparts (−1.58 μV) [*F*(1, 50) = 6.05, *p* = 0.017, η_p_^2^ = 0.11] (see Supplementary Figs S2 and S6). Furthermore, there was a marginally significant interaction of hemisphere × MA [*F*(2, 100) = 3.07, *p* = 0.051, η_p_^2^ = 0.06]. Post hoc tests revealed larger N1 amplitude in HMA individuals (−3.40 μV) than that in their LMA peers (−1.77 μV) at right sites [*F*(1, 50) = 4.052, *p* = 0.050, η_p_^2^ = 0.08] (see Fig. 3).

*P2*. **For latency**, no significant effects were observed. **For amplitude**, an interaction of area × MA reached the level of significance [*F*(2, 100) = 9.61, *p* = 0.000, η_p_^2^ = 0.16]. HMA group elicited significant greater P2 amplitude at frontal sites (6.22 μV) and frontal-central sites (5.72 μV) than those at central sites (5.01 μV) [*F*(2, 49) = 14.64, *p* = 0.000, η_p_^2^ = 0.37].

*P3b*. **For latency**, the four-way rmANCOVA yielded a significant main effect of AS [*F*(1, 50) = 4.78, *p* = 0.033, η_p_^2^ = 0.09], indicating that the LAS group (418.28 ms) had longer P3b latency than HAS group (390.15 ms), and significant interactions of area × AS [*F*(2, 100) = 3.46, *p* = 0.035, η_p_^2^ = 0.07], AS × MA [F(1, 50) = 4.32, *p* = 0.043, η^2^ = 0.08], and area × hemisphere × MA [*F*(4, 200) = 2.78, *p* = 0.028, η_p_^2^ = 0.05]. Among the LAS group, the P3b latency in HMA individuals (438.12 ms) was longer than that in their LMA peers (398.43 ms) [*F*(1, 50) = 4.63, *p* = 0.036, η_p_^2^ = 0.08]. Among HMA individuals, the LAS group (438.12 ms) had longer P3b latency than the HAS group (383.44 ms) [*F*(1, 50) = 9.03, *p* = 0.004, η_p_^2^ = 0.15] (see Supplementary Figs S3 and S7). Additionally, compared to the LMA group (382.72 ms), the HMA group (424.09 ms) showed longer P3b latency at the O1 site [*F*(1, 50) = 6.05, *p* = 0.017, η_p_^2^ = 0.11] (see Fig. 3). Furthermore, there were also marginally significant interactions of area × MA [*F*(2, 100) = 2.60, *p* = 0.079, η_p_^2^ = 0.05] and area × AS × MA [*F*(2, 100) = 2.90, *p* = 0.060, η_p_^2^ = 0.06]. **For amplitude**, the main effect of area [*F*(1.46, 73.15) = 3.21, *p* = 0.061, η_p_^2^ = 0.06], with more positive amplitudes at parietal sites (8.83 μV) and parietal-occipital sites (8.70 μV), and interactions of hemisphere × AS × MA [*F*(2, 100) = 3.09, *p* = 0.050, η_p_^2^ = 0.06], area × hemisphere [*F*(4, 200) = 3.84, *p* = 0.005, η_p_^2^ = 0.07] and area × hemisphere × AS [*F*(4, 200) = 3.56, *p* = 0.008, η_p_^2^ = 0.07] reached significance. While LMA individuals performed tasks, greater P3b amplitude in HAS group (9.49 μV) were found than that in LAS group (7.06 μV) at left sites [*F*(1, 50) = 4.04, *p* = 0.050, η_p_^2^ = 0.08] (see Supplementary Figs S4 and S7).

#### **High arithmetic skill enhanced the processing of the HMA group in the calculation task**

*N1*. **For latency**, analysis revealed a significant interaction of area × AS [*F*(2, 100) = 3.96, *p* = 0.022, η_p_^2^ = 0.07]. Among the HAS group, the latencies at the frontal (105.96 ms) and frontal-central sites (105.31 ms) were longer than that at central sites (101.32 ms) [*F*(2, 49) = 6.50, *p* = 0.003, η_p_^2^ = 0.21]. **For amplitude**, a main effect of hemisphere [*F*(1.71, 85.25) = 4.86, *p* = 0.014, η_p_^2^ = 0.09] and interaction of area × MA × AS [*F*(2, 100) = 5.74, *p* = 0.004, η_p_^2^ = 0.103] reached the level of significance. Post hoc tests revealed no significant group difference (see Supplementary Fig. S6).

**Figure 4:**
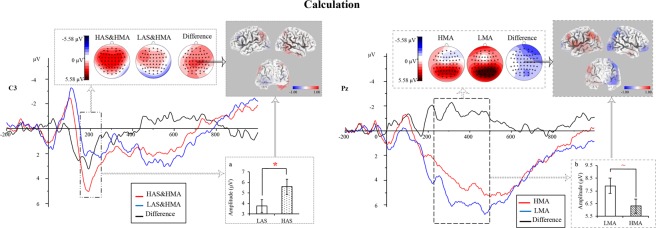
Grand-average waveforms at the C3 and Pz sites, and scalp topographies and source localization of group difference in the calculation task. Left: N1 Difference for the HAS&HMA group and the LAS&HMA group at the C3 electrode. As suggested, in the HMA group, HAS individuals had a more positive amplitude than their LAS peers. The difference was the amplitude of HAS&HMA minus that of LAS&HMA. The sLORETA analysis of P2 suggested that among HMA in the calculation task, enhanced activation in the right SPL, IPL, and precuneus of the right parietal lobe was observed in HAS. Right: Difference waveform for P3b between the HMA group and LMA group. As suggested, compared with the HMA group, amplitudes of the LMA group were more positive. The difference was obtained by amplitudes of the HMA group minus those of the LMA group. The sLORETA analysis of P3b revealed that HMA led to a significantly enhanced activation in the left supramaginal gyrus, IPL, and precentral gyrus, and decreased activation in the right SPL and IPL. (**a**) Group difference of AS in P2 amplitude for HMA individuals. (**b**) P3b difference of MA. Additionally, the scalp topography of the P2 and P3b components shows the interference effect in the 150–250 and 250–500 ms window respectively. *p < 0.05; ^~^*p* < 0.1.

*P2*. **For latency**, there was a marginally significant interaction of hemisphere × AS [*F*(2, 100) = 2.72, *p* = 0.071, η_p_^2^ = 0.05] and interaction of area × hemisphere × AS × MA (*F*(4, 200) = 2.12, *p* = 0.079, η_p_^2^ = 0.04). In addition, among HMA individuals, the latencies of P2 in HAS group (186.08 ms) were shorter compared with that in LAS group (211.70 ms) at the F3 electrode [*F*(1, 50) = 6.64, *p* = 0.013, η_p_^2^ = 0.12] (see Supplementary Figs S5 and S6). **For amplitude**, the interaction of area × hemisphere × MA × AS [*F*(4, 200) = 2.29, *p* = 0.061, η_p_^2^ = 0.04] reached significance. Post hoc tests revealed that for HMA individuals, HAS group (5.59 μV) had a larger P2 amplitude than LAS group (3.76 μV) at the C3 electrode [*F*(1, 50) = 4.03, *p* = 0.050, η_p_^2^ = 0.08] (see Fig. 4; Supplementary Fig. S6).

Standardized low resolution brain electromagnetic tomography (sLORETA) is a method that computes images of electric neuronal activity from EEG and MEG^36^. This was done because significant differences in MA were found in the present study. By selecting only three instead of all time samples statistically significant in the P2 time range, this analysis was made more conservative. The sLORETA analysis revealed that this difference between HAS&HMA and LAS&HMA was associated with activation differences in SPL (superior parietal lobule, BA 7), IPL (BA 40), and the precuneus of the right parietal lobe (BA 7) (see Fig. 4).

*P3b*. **For latency**, There was a marginally significant main effect of AS [*F*(1, 50) = 3.24, *p* = 0.078, η_p_^2^ = 0.06], and marginally significant interactions of area × hemisphere [*F*(3.30, 164.87) = 2.49, *p* = 0.057, η_p_^2^ = 0.05] and hemisphere × AS [*F*(2, 100) = 2.83, *p* = 0.064, η_p_^2^ = 0.05]. Post hoc tests revealed a shorter P3b latency in HAS group (380.47 ms) than that in LAS group (415.82 ms) at middle sites [*F*(1, 50) = 5.21, *p* = 0.027, η_p_^2^ = 0.09]. **For amplitude**, the main effect of MA [*F*(1, 50) = 3.24, *p* = 0.088, η_p_^2^ = 0.06], with more positive P3b amplitude in LMA group (7.89 μV) (see Fig. 4), and AS [*F*(1, 50) = 2.99, *p* = 0.090, η_p_^2^ = 0.06], with more positive P3b amplitude in HAS group (7.76 μV), reached significance marginally. The sLORETA analysis revealed that this difference between HMA group and LMA group was associated with activation differences in the supramaginal gyrus (BA 40) and IPL (BA 40) of the left parietal lobe, SPL (BA 7) and IPL (BA 40) of the right parietal lobe, and the precentral gyrus (BA 44) of the left frontal lobe (see Fig. 4).

#### **Neural-behavioral correlation**

**Figure 5:**
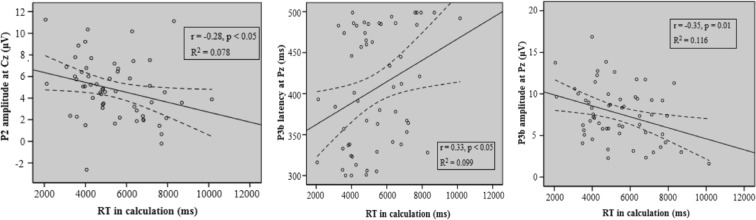
Correlations between neural and behavioral findings. Left: A significant positive correlation between P2 amplitude at the Cz site and RT in the calculation task. Middle and right: For P3b, there was a positive correlation between the P3b latency and RT, and a negative correlation between the P3b amplitude and RT at the Pz site in the calculation task. As suggested, the longer latency and smaller amplitude will result in a longer RT, indicating that in complex tasks, processing speed affects RT. In addition, the more resources allocated, the shorter the RT was. *r* = Pearson’s correlation coefficient.

Correlation analyses were conducted between RT, ACC, and flawed score and the significant EEG findings to explore the relationship between neural and behavioral findings with test anxiety, state anxiety and trait anxiety as covariates. P2 amplitude at the Cz site (*r*(52) = −0.28, *p* = 0.044, Bonferroni *p* > 0.05) were significantly correlated with RT in the calculation task (see Fig. 5), but this negative relationship did not survive after correction. Moreover, RT in the calculation task was significantly correlated with P3b latency (*r*(52) = 0.33, *p* = 0.016, Bonferroni *p* < 0.05) and amplitude (*r*(52) = −0.35, *p* = 0.010, Bonferroni *p* < 0.05) over the Pz electrode (see Fig. 5).

## Discussion

An initial objective of the present study was to explore the link between MA and performance and to investigate the potential roles of AS on this link behaviorally and psychophysiologically. The main findings could be summarized as follows: The HAS group performed more accurately and faster than LAS group; In the comparison task, compared with LMA peers, HMA individuals elicited shorter latencies and larger amplitude of N1 and longer P3b latency, and a larger N1 amplitude was found in the individuals with HMA than those with LMA while HAS individuals performed comparison task; In the calculation task, results showed shorter P2 latency and larger P2 amplitude in HAS group than LAS group among HMA individuals.

### **Effects of high arithmetic skill in behavioral**

The most obvious findings to emerge from the analyses were the main effects of AS in the calculation task. For RT, ACC and flawed score, however, no evidence was detected of an effect on the relationship between MA and performance. This result is in accordance with some recent studies^8,18–20,37,38^, indicating that no effect of MA was found, at least in performance effectiveness. Suárez-Pellicioni *et al*. (2013) and Basten *et al*. (2012) suggested that RT represents the outcome of processing, not processing itself, and should not be considered a measure of processing efficiency^19,39^. According to attentional control theory, anxiety may not impair performance effectiveness (e.g., accuracy) when it leads to the use of compensatory strategies^17^. In the present study, the findings indicated that compared to MA (negative emotion about math stimuli), AS (basic mathematical ability) showed a greater effect on performance. In other words, ability is a more important factor for performance. However, as a valuable way of assessing processing efficiency, neuroimaging was adopted to reveal the underpinnings of the effect of MA. In early ERP components (N1 and P2) and late components (P3b), the effects of MA and the role of AS were determined in the present study.

### **Deficits of math anxiety in the comparison task**

Compared with LMA individuals, more negative amplitudes of N1 in their HMA peers were found in the comparison task, consistent with Chang *et al*. (2017). These scholars revealed that more fronto-parietal attention network was activated in HMA individuals compared to LMA individuals and suggested that simple arithmetic was not simple for HMA individuals^20^. Enhanced amplitudes of N1 indicate discrimination of attended stimuli and/or more attentional resources^40–42^. As early visual processing, N1 is related to the early processing of stimuli and are sensitive to the physical properties of the stimuli^40^. It also indexes the orienting of attention^43^. Visuospatial orienting of attention that enhanced the stimulus-evoked neural activity reflected in enhanced amplitudes of the N1 component of ERP. Bar-Haim *et al*. (2005) investigated attention bias in anxiety with an attention-shifting paradigm, and they found that compared to individuals with low anxiety, threat-related faces elicited faster latencies and greater amplitudes of early ERP components in those with high anxiety^41^. MA, a negative emotional reaction to dealing with numbers, may consume the attentional resources required for the task. Hence, individuals with HMA need more attentional resources to discriminate numbers at the early stage. According to attentional control theory, Eysenck *et al*. (2007) suggested anxiety would increase stimulus-driven bottom-up processing^17^. In the present study, individuals with HMA showed shorter N1 latency. Furthermore, for individuals with HAS, numbers elicited larger amplitudes of N1 in the HMA group than in the LMA group in the comparison task. Therefore, we inferred that for the HAS group, HMA can enhance the ability to distinguish attended stimuli compared to LMA.

In the present study, students with HMA elicited a longer latency of P3b in the comparison task relative to those with LMA, consistent with previous findings^44^. Suárez-Pellicioni *et al*. (2013) have explored the effect of MA on the performance of simple arithmetic processing and found large-split solutions elicited a more enhanced and delayed P3b in the highly math-anxious group^19^. They proposed that these individuals had difficulty in inhibiting the extended processing of irrelevant information. As one of the late components, P3b is related to the classification of stimuli and extraction of mental arithmetic facts^21,45,46^. The latency of P3b is related to the stimulus evaluation time, or more generally to the speed of cognitive processing^47–49^. The result in the present study indicated that individuals with HMA need more time to evaluate stimuli, so their processing speed was slower, which also verified the results of Chang *et al*. (2017) that a simple problem was not simple for those with HMA^20^. However, an interesting result was observed. For individuals with high math anxiety, numbers elicited shorter latencies of P3b in the HAS group than that in the LAS group in the comparison task. Therefore, we inferred that for the HMA group, AS could enhance evaluation time.

### **Enhancements of high arithmetic skill for the HMA group in the calculation task**

In numerical cognition, early ERP components, such as P2, are evoked by mental calculation and other mental processes, such as counting^21^. The P2 component in numerical processing is related to attentional resources consumed by negative stimuli and to the involvement of attention in perceptual processing^18^^,^^23^. This positive component at the anterior scalp indicates processing of attention^50,51^. Larger amplitudes of P2 may reflect the inhibition of irrelevant stimuli and ability to focus on targeted stimuli. Salillas and Carreiras (2014) suggested an effect at approximately 200 ms for the automatic analysis of numerical magnitude^52^. In the present study, the amplitude of individuals with HAS was more positive than those with LAS while the HMA group performed the calculation task, indicating a better inhibitory control in HAS individuals. In addition, P2 amplitude was negatively correlated with shorter reaction time in the calculation task, which suggested that based on facilitated discrimination and a more precise encoding stage, arithmetic skill could improve the timing of working memory processes and performance^43^. Analysis of the source of this difference showed enhanced activation in the SPL, IPL, and precuneus of the right parietal lobe (see Fig. 4). According to the triple-code model, SPL, a region of the parietal area, is associated with domain-general attention processes related to numerical processing^53^. This finding showed that HAS engaged more demands on the domain-general support area, and more attentional resources were allocated to numerical processes.

A result fascinating us is that, in the calculation task, smaller amplitudes were observed in the HMA group. The amplitude of P3b is linked to the amount of attention allocated to stimuli^44,48,49,54^. However, this depends on task difficulty. If complexity exceeds attention resources, this amplitude will decrease as task difficulty increases^55^. The calculation task adopted in the present study was two-digit multiplication, which may be too difficult. This requires further research to demonstrate. Source localization of this group effect in the current study showed differences in activation in the bilateral IPL, the supramarginal gyrus of the left parietal lobe, the right SPL, and the left precentral gyrus (see Fig. 4). IPL, supramaginal gyrus, and SPL belong to the parietal lobe. During multiplication, the results they have obtained are through verbal computations, so the left parietal region would be strongly activated. Hartwright *et al*. (2018) discovered the reduced activation of the right SPL, which supported domain-general attention processes related to numerical processing^56^. In our study, the reduced activation of the right SPL in individuals with HMA indicated that their worse performance may be due to the lesser demands on domain-general-supporting areas or that fewer resources are available to high math anxious people. Although many studies supported a special role of the IPL in arithmetic processing^57,58^, Lyons *et al*. (2012) did not find an effect of MA in the bilateral IPL^29^. Further studies need to investigate the role of IPL on the neural basis of MA.

Neural-behavioral correlation in our study showed that the amplitude of P3b was negatively correlated with RT, and the latency of P3b was positively correlated with RT. These findings indicated that longer RT may result from longer stimulus evaluation time spent and fewer attentional resources consumed.

This study has several rather remarkable findings: First, individuals with HMA need more attentional resources to discriminate numbers at the early stage, which enhanced the evaluation time at the late stage in the comparison. They proved that a simple problem was not simple for those with HMA. Then, AS enhanced the ability to encode numbers more precisely, inhibit unrelated stimuli and focus on a target in the complex task. It showed a positive effect of HAS for HMA. These findings may shed light on the link between MA and performance and help us find new ways to intervene against the negative effects of MA. Due to the small sample size of this study, our results must be interpreted with caution.

## Conclusions

According to the findings in the present study, no evidence of a math anxiety–performance link was determined behaviorally. However, more occupied attentional resources and an enhanced evaluation time were found at the early and late stage, respectively, while individuals with HMA performed the comparison task. Furthermore, arithmetic skill enhanced the performance efficiency of individuals with HMA, especially in a complex task. In addition, source analysis showed the activation of parietal regions in arithmetic processing. These results suggest mathematical educational interventions would be more effective when considering differences between populations.

## Supplementary information

Supplementary materials

## Data Availability

The datasets analyzed during the present study are available from the corresponding author on reasonable request.
