# Supplementary material for: Arithmetic Skill May Refine the Performance of Individuals with High Math Anxiety, Especially in the Calculation Task: An ERP Study
Source: Sci Rep. 2019 Sep 16;9:13283. doi: 10.1038/s41598-019-49627-7 (PMC6746767; doi:10.1038/s41598-019-49627-7)
Supplement: Supplementary file 1 — Supplementary materials [file 41598_2019_49627_MOESM1_ESM.docx]

**Arithmetic Skill May Refine the Performance of Individuals with High Math Anxiety, Especially in the Calculation Task: An ERP Study**

Bijuan Huang^1^, Hongxia Li^1^, Weixing Yang^1^, Shuang Cui^1^, Yaru Gao^1^, Jiwei Si^1^*

1) School of Psychology, Shandong Normal University, Jinan, China

Note: * Corresponding Author: Prof. Jiwei Si, School of Psychology, Shandong Normal University, No.1500, University Road, University Technology Garden, Changqing District, Jinan, China. E-mail: sijiwei1974@126.com

The number of figures is 8, and the number of tables is 5.

**Supplementary materials: Tables and figures**

**Supplementary Table S1 Correlations between math anxiety, test anxiety, state anxiety and trait anxiety.**

|  | 1 | 2 | 3 | 4 | 5 |
| --- | --- | --- | --- | --- | --- |
| 1. AS | - | -0.09 | 0.17 | 0.14 | 0.19 |
| 2. MA | -0.09 | - | 0.46** | 0.31* | 0.39** |
| 3. TAS | 0.17 | 0.46** | - | 0.50** | 0.58** |
| 4. STAI-S | 0.14 | 0.32* | 0.51** | - | 0.84** |
| 5. STAI-T | 0.19 | 0.36** | 0.56** | 0.86** | - |

Note. AS: arithmetic skill; MA: math anxiety; TAS: test anxiety; STAI-S: state anxiety; STAI-T: trait anxiety. Coefficient under the diagonal line is controlled by gender, and coefficient above the diagonal line is the coefficient of zero-order correlation. * *P* < 0.05; ** *P* < 0.01.

**Supplementary Table S2 Mean and standard deviation of RT, ACC and flawed scores for different levels of arithmetic skill and math anxiety.**

| Dependent  variables Complexity | | LAS | | HAS | |
| --- | --- | --- | --- | --- | --- |
|  |  | LMA | HMA | LMA | HMA |
| RT  (ms) | Comparison | 1275.76±511.61 | 1184.33±479.89 | 918.87±240.38 | 870.40±271.32 |
|  | Calculation | 5922.56±1393.49 | 5967.31±1925.68 | 4894.10±1638.99 | 4585.86±1532.51 |
| ACC  (%) | Comparison | 95.00±5.65 | 96.08±3.92 | 97.40±2.31 | 97.00±3.37 |
|  | Calculation | 87.86±6.31 | 85.92±5.50 | 91.92±3.70 | 90.17±6.35 |
| Flawed  scores (%) | Comparison | 8.39±5.40 | 8.00±4.27 | 6.35±3.25 | 7.92±4.83 |
|  | Calculation | 15.89±5.15 | 16.50±5.49 | 10.77±4.10 | 13.67±6.59 |

Note. LAS: Low arithmetic skill; HAS: High arithmetic skill; LMA: Low math anxiety; HMA: High math anxiety; RT: Reaction time of correct responses (in ms); ACC: Accuracy (in %). Flawed scores: The sum of proportion of errors and proportion of the number of extreme RT values (in %). The values of covariates: State anxiety = 35.65, trait anxiety = 39.56, and test anxiety = 15.56.

**Supplementary Table S3 ANOVA of RT, ACC and flawed scores for comparison and calculation task.**

| Source | Dependent | *SS* | *df* | *MS* | *F* | *P* | *η_p_^2^* |
| --- | --- | --- | --- | --- | --- | --- | --- |
| Intercept | RT in comparison | 1728444.577 | 1 | 1728444.577 | 10.632 | .002 | .175 |
|  | RT in calculation | 43009851.515 | 1 | 43009851.515 | 15.219 | .000 | .233 |
|  | ACC in comparison | 19510.116 | 1 | 19510.116 | 1189.932 | .000 | .960 |
|  | ACC in calculation | 14023.060 | 1 | 14023.060 | 431.693 | .000 | .896 |
|  | flawedscore in comparison | 47.352 | 1 | 47.352 | 2.340 | .132 | .045 |
|  | flawedscore in comparison | 485.439 | 1 | 485.439 | 15.805 | .000 | .240 |
| STAIT1 | RT in comparison | 140090.286 | 1 | 140090.286 | .862 | .358 | .017 |
|  | RT in calculation | 260040.914 | 1 | 260040.914 | .092 | .763 | .002 |
|  | ACC in comparison | 9.639 | 1 | 9.639 | .588 | .447 | .012 |
|  | ACC in calculation | 29.751 | 1 | 29.751 | .916 | .343 | .018 |
|  | flawedscore in comparison | 1.946 | 1 | 1.946 | .096 | .758 | .002 |
|  | flawedscore in comparison | 39.522 | 1 | 39.522 | 1.287 | .262 | .025 |
| STAIT2 | RT in comparison | 153738.043 | 1 | 153738.043 | .946 | .335 | .019 |
|  | RT in calculation | 37673.785 | 1 | 37673.785 | .013 | .909 | .000 |
|  | ACC in comparison | .053 | 1 | .053 | .003 | .955 | .000 |
|  | ACC in calculation | 8.763 | 1 | 8.763 | .270 | .606 | .005 |
|  | flawedscore in comparison | 39.565 | 1 | 39.565 | 1.955 | .168 | .038 |
|  | flawedscore in comparison | 29.065 | 1 | 29.065 | .946 | .335 | .019 |
| TAI | RT in comparison | 70041.786 | 1 | 70041.786 | .431 | .515 | .009 |
|  | RT in calculation | 307820.985 | 1 | 307820.985 | .109 | .743 | .002 |
|  | ACC in comparison | 2.452 | 1 | 2.452 | .150 | .701 | .003 |
|  | ACC in calculation | 4.070 | 1 | 4.070 | .125 | .725 | .002 |
|  | flawedscore in comparison | 30.144 | 1 | 30.144 | 1.490 | .228 | .029 |
|  | flawedscore in comparison | .987 | 1 | .987 | .032 | .858 | .001 |
| MA | RT in comparison | 91689.246 | 1 | 91689.246 | .564 | .456 | .011 |
|  | RT in calculation | 771567.020 | 1 | 771567.020 | .273 | .604 | .005 |
|  | ACC in comparison | 12.161 | 1 | 12.161 | .742 | .393 | .015 |
|  | ACC in calculation | 59.272 | 1 | 59.272 | 1.825 | .183 | .035 |
|  | flawedscore in comparison | 2.428 | 1 | 2.428 | .120 | .731 | .002 |
|  | flawedscore in comparison | 30.623 | 1 | 30.623 | .997 | .323 | .020 |
| AS | **RT in comparison** | **1523890.309** | **1** | **1523890.309** | **9.374** | **.004** | **.158** |
|  | **RT in calculation** | **20904687.197** | **1** | **20904687.197** | **7.397** | **.009** | **.129** |
|  | ACC in comparison | 50.425 | 1 | 50.425 | 3.075 | .086 | .058 |
|  | **ACC in calculation** | **205.967** | **1** | **205.967** | **6.341** | **.015** | **.113** |
|  | flawedscore in comparison | 19.839 | 1 | 19.839 | .980 | .327 | .019 |
|  | **flawedscore in comparison** | **214.001** | **1** | **214.001** | **6.968** | **.011** | **.122** |
| MA * AS | RT in comparison | 574.071 | 1 | 574.071 | .004 | .953 | .000 |
|  | RT in calculation | 705889.914 | 1 | 705889.914 | .250 | .619 | .005 |
|  | ACC in comparison | 3.310 | 1 | 3.310 | .202 | .655 | .004 |
|  | ACC in calculation | .283 | 1 | .283 | .009 | .926 | .000 |
|  | flawedscore in comparison | 16.020 | 1 | 16.020 | .792 | .378 | .016 |
|  | flawedscore in comparison | 19.664 | 1 | 19.664 | .640 | .427 | .013 |
| Error | RT in comparison | 8128170.080 | 50 | 162563.402 |  |  |  |
|  | RT in calculation | 141300752.268 | 50 | 2826015.045 |  |  |  |
|  | ACC in comparison | 819.799 | 50 | 16.396 |  |  |  |
|  | ACC in calculation | 1624.193 | 50 | 32.484 |  |  |  |
|  | flawedscore in comparison | 1011.720 | 50 | 20.234 |  |  |  |
|  | flawedscore in comparison | 1535.695 | 50 | 30.714 |  |  |  |
| Total | RT in comparison | 74516375.997 | 57 |  |  |  |  |
|  | RT in calculation | 1794294141.125 | 57 |  |  |  |  |
|  | ACC in comparison | 530156.250 | 57 |  |  |  |  |
|  | ACC in calculation | 452257.813 | 57 |  |  |  |  |
|  | flawedscore in comparison | 4498.438 | 57 |  |  |  |  |
|  | flawedscore in comparison | 13506.250 | 57 |  |  |  |  |

Note. SS: Sum of squares; MS: Mean squares; df: Degree of freedom; η_p_^2^: Parietal Eta Square. The values of covariates: State anxiety = 35.65, trait anxiety = 39.56, and test anxiety = 15.56.

**Supplementary Table S4 Repeated measure ANOVA of ERPs for comparison and calculation task.**

|  | N1-Latency | N1-Amplitude | P2-Latency | P2-Amplitude | P3b-Latency | P3b-Amplitude |
| --- | --- | --- | --- | --- | --- | --- |
| Variables | (-, 80~120) | | (+, 150~250) | | (+, 250~500) | |
| Area |  |  |  |  |  | (~) |
| Area*MA |  |  |  | (+) | (~) |  |
| Area*AS | + |  |  |  | (+) |  |
| Area*MA*AS |  | + |  |  | (~) | (+) |
| Hemi |  | + / (+) |  |  |  |  |
| Hemi*MA |  | (~) |  |  |  |  |
| Hemi*AS |  |  | ~ |  | ~ |  |
| Hemi*MA*AS |  |  |  |  |  |  |
| Area*Hemi | (+) | (+) |  |  | ~ | (+) |
| Area*Hemi*MA |  |  |  |  | (+) |  |
| Area*Hemi *AS |  |  |  |  |  | (+) |
| Area*Hemi* MA*AS |  |  | ~ | ~ |  |  |
| MA | (+) |  |  |  |  | ~ |
| AS |  |  |  |  | ~ / (+) | ~ |
| MA*AS |  | (+) |  |  | (+) |  |

(+): Significant in the comparison task; +: Significant in the calculation task; (~): Marginally significant in the comparison task; ~: Marginally significant in the calculation task; The values of covariates: State anxiety = 35.65, trait anxiety = 39.56, and test anxiety = 15.56.

**Supplementary Figure S1.**

left

right

HEOG / VEOG

FCz

The distribution of 62 electrodes. The black points with red circle are the 18 electrodes chosen to analyze.

**Supplementary Figure S2.**


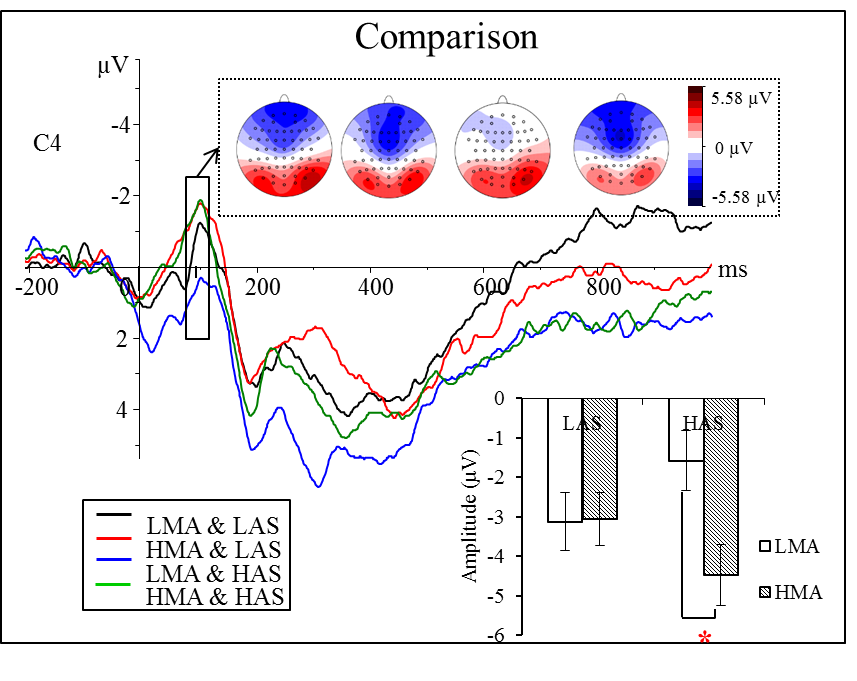


Grand-average waveforms and scalp topographies for four groups at the C4 site in the comparison task. As suggested, for HAS individuals, amplitudes of the HMA group were more negative than those of the LMA group. Line chart in the bottom: Group difference in N1 amplitude in the comparison task. The values of covariates: State anxiety = 35.65, trait anxiety = 39.56, and test anxiety = 15.56.

**Supplementary** **Figure S3**.

When HMA individuals performed the comparison task, latencies of the LAS group > that of the HAS group; For LAS individuals, latencies of the LMA group < that of the HMA group. * *P* < 0.05. Error bar indicates standard error.

**Supplementary Figure S4.**

For LMA group, amplitude of LAS individuals < that of HAS peers at left sites in the comparison task. Error bar indicates standard error.

**Supplementary Figure S5.**

At the F3 site, LAS&HMA individuals showed longer latency compared with HAS&HMA peers in the calculation task. * *P* < 0.05. Error bar indicates standard error.

**Supplementary Figure S6.**


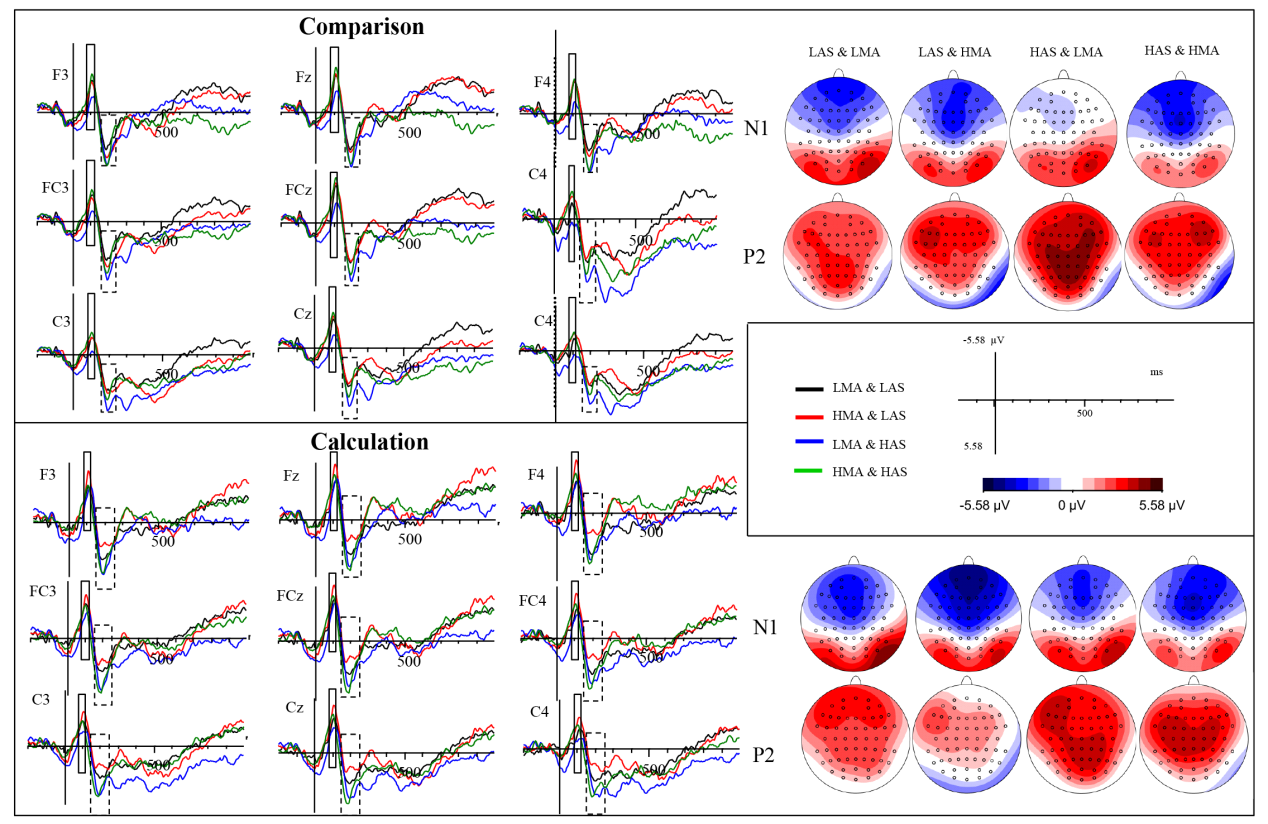


Grand-average waveforms and scalp topographies of N1 and P2 for all four groups in the comparison task and calculation task. The values of covariates: State anxiety = 35.65, trait anxiety = 39.56, and test anxiety = 15.56.

**Supplementary Figure S7.**


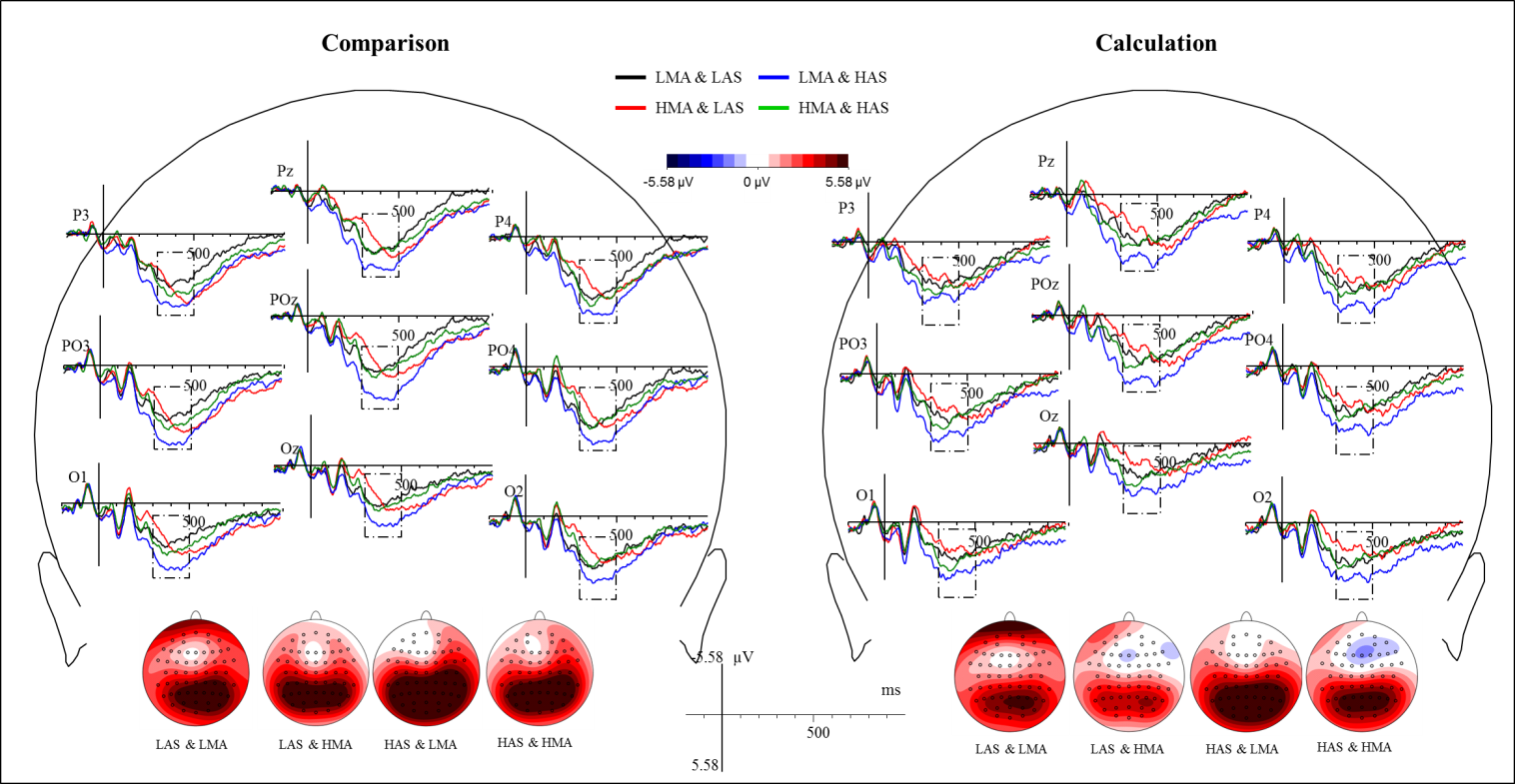


Grand-average waveforms and scalp topographies of P3b for all four groups in the comparison task and calculation task. The values of covariates: State anxiety = 35.65, trait anxiety = 39.56, and test anxiety = 15.56.

**Supplementary Table S5. The operations in the calculation task**

| The first operand | The second operand | Solutions | The first operand | The second operand | Solutions |
| --- | --- | --- | --- | --- | --- |
| 92 | 3 | 262 | 72 | 49 | 4176 |
| 57 | 6 | 372 | 34 | 81 | 2482 |
| 42 | 4 | 148 | 87 | 42 | 2872 |
| 91 | 6 | 576 | 57 | 64 | 3248 |
| 96 | 3 | 302 | 92 | 56 | 5888 |
| 94 | 2 | 198 | 86 | 19 | 2236 |
| 57 | 2 | 104 | 27 | 46 | 1432 |
| 21 | 6 | 156 | 74 | 56 | 4736 |
| 62 | 8 | 456 | 83 | 62 | 4398 |
| 49 | 4 | 176 | 48 | 26 | 1680 |
| 98 | 6 | 558 | 39 | 42 | 1326 |
| 54 | 2 | 118 | 43 | 52 | 1850 |
| 84 | 9 | 710 | 89 | 32 | 2226 |
| 84 | 7 | 624 | 53 | 28 | 1908 |
| 71 | 2 | 152 | 21 | 48 | 1155 |
| 97 | 2 | 204 | 37 | 58 | 2442 |
| 46 | 9 | 458 | 74 | 52 | 3256 |
| 68 | 7 | 512 | 86 | 41 | 2924 |
| 98 | 4 | 372 | 89 | 68 | 6586 |
| 56 | 8 | 488 | 24 | 91 | 2016 |
| 42 | 3 | 142 | 56 | 83 | 4144 |
| 73 | 2 | 136 | 47 | 62 | 2492 |
| 24 | 8 | 232 | 57 | 26 | 1937 |
| 76 | 8 | 568 | 27 | 94 | 2349 |
| 62 | 9 | 514 | 23 | 48 | 1288 |
| 39 | 4 | 136 | 17 | 68 | 1258 |
| 84 | 2 | 158 | 98 | 42 | 3430 |
| 23 | 6 | 108 | 64 | 87 | 6080 |
| 42 | 6 | 282 | 89 | 26 | 2937 |
| 72 | 3 | 201 | 63 | 24 | 1134 |
| 63 | 2 | 136 | 41 | 56 | 2664 |
| 26 | 8 | 168 | 64 | 19 | 1600 |
| 54 | 7 | 342 | 76 | 34 | 2128 |
| 78 | 2 | 166 | 62 | 31 | 1550 |
| 98 | 7 | 652 | 84 | 37 | 3696 |
| 29 | 4 | 136 | 47 | 36 | 2114 |
| 48 | 7 | 372 | 61 | 24 | 1098 |
| 79 | 2 | 148 | 82 | 94 | 7052 |
| 78 | 3 | 248 | 72 | 94 | 6336 |
| 56 | 9 | 458 | 28 | 59 | 1876 |

Problems in the calculation task were all multiplication. The first operand was double-digit (17~98), but the second operand was double-digit (19~94) or single-digit (2~9). 600 operations were randomly generated. The second operand of half of multiplication problems was single-digit, and the other half was double-digit. The criterion for randomly choosing operands of arithmetic verification problems were as follows: a. No operand contained the identical tens digit or single digit (e.g., 24 * 27 or 34 * 54); b. no operand contained 5 or 0 (e.g., 30 or 35); c. Three or four numbers of the two operands are different (e.g., 3 * 26 or 24 * 53); d. no results were double-digit when the one operand was single-digit, and no was three-digit when the both operands were double-digit. (e.g., 3 * 12 or 12 * 34). In arithmetic verification task, the solutions were generated as followed: Firstly, a random set of single-digit (-6~-9 or 6~9) was generated; Then, the first operand plus that single-digit; and the first operand was multiplied by the single-digit to produce the solutions in arithmetic verification task. The solutions were added or minus 1 to keep the parity of the results (Hinault, Dufau, & Lemaire, 2014) (118~710; 1098~7052).


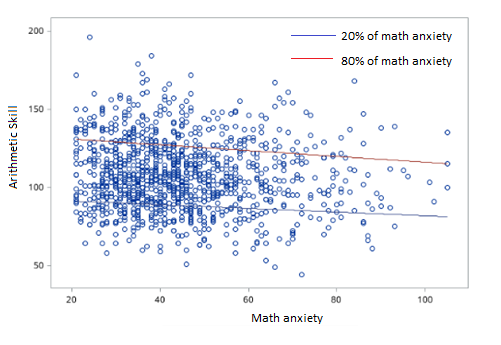


**Supplementary** **Figure S8.** the results of a pre-measure. Regression analysis of 20% and 80% on RMARS. Low math anxiety group: ≤20% on RMARS; and high math anxiety group: ≥80% on RMARS.
